# Microwave support of the alcoholic fermentation process of cyanobacteria *Arthrospira platensis*

**DOI:** 10.1007/s11356-019-05427-0

**Published:** 2019-05-23

**Authors:** Anna Nowicka, Marcin Zieliński, Marcin Dębowski

**Affiliations:** Department of Environment Engineering, Warszawska 117a, 10-720 Olsztyn, Poland

**Keywords:** Ethanol production, *Arthrospira platensis*, Pretreatment, Thermal hydrolysis, Microwave

## Abstract

The search for a balance between the energy-related challenges of the future and providing nutritional security has resulted in the development of a market for biofuels of successive generations. The larger their portion in biofuel production, the less the prices of agricultural products will increase. The use of algae, cyanobacteria and aquatic plants in the production of liquid fuels is an alternative. The aim of this study was to determine the effect of thermal hydrolysis on degradation of polysaccharides contained in biomass of cyanobacteria *Arthrospira platensis* and to assess the effectiveness of ethanol production from preconditioned biomass. The study is aimed at the selection of the most advantageous parameters of thermochemical hydrolysis to reach the experiment variant with the best effects, degree of polysaccharide degradation and effectiveness of alcohol fermentation. The experiment was divided into two stages; in stage I, the possibility of obtaining fermentable sugars by hydrothermal and chemical treatment of the substrate was tested. Stage II involved an assessment of the effectiveness of the pretreatment methods to produce bioethanol in alcohol fermentation. Yeast used in industrial ethanol production—*Saccharomyces cerevisiae* As4—was used in the alcohol fermentation. The results have shown that the temperature of 150 °C was the most beneficial for the process of thermohydrolysis, and the mash in the microwave-heated sample contained the highest concentration of alcohol (0.97 g/l), which is 98% more than in the control mash and 37% more than in the conventionally heated sample.

## Introduction

The term “bioethanol” denotes ethanol at a concentration over 99.8% obtained from plant material (Schacht et al. 2008). Hopes are pinned on bioethanol that it will solve the problem of the use of fossil fuels; it is regarded as a product which will not only eliminate the energy crisis but which will have a positive impact on the deteriorating natural environment (Aditiya et al. 2016). The global output of ethanol in 1998 amounted to 31.2 billion dm^3^, to increase to approx. 50 billion dm^3^ in 2006 (Sybirny et al. 2007). The production output was over 100 billion dm^3^ in 2011, and its annual increase by 3–7% was expected for the 2012–2015 period (Aditiya et al. 2016). The USA and Brazil are the largest global producers of bioethanol, with such positions in Europe being occupied by Spain and France. According to forecasts, the RES (renewable energy sources) consumption in Poland will have increased by 2020, from 1 to 2.5% to over 14% (Sybirny et al. 2007). Bioethanol is not a new energy source; it was widely used in the early twentieth century in many European countries and in the USA, but it was replaced with petrol due to high costs. The 1970 oil crisis returned attention to bioethanol; studies have shown that the use of this fuel has a beneficial effect on combustion engines; its advantages include a high octane number, improvement of the oxidising properties and reduction of toxic emissions (Krylova et al. 2008, Balat 2011).

Development of renewable energy sources is associated with the concept of the third generation fuels, which include algae and aquatic plants; according to numerous studies, aquatic biomass can yield biogas, bioethanol and biodiesel.

**Fig. 1:**
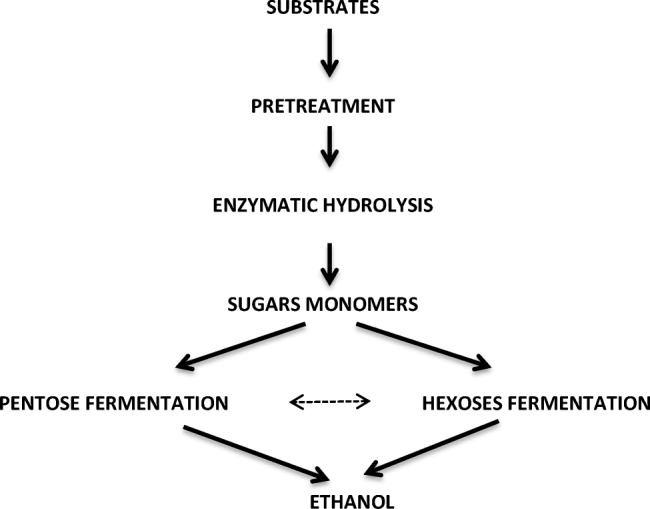
The general diagram of converting biomass to bioethanol

Increasing the efficiency of alcohol fermentation of biomass requires both an effective method of their pretreatment, the right composition of enzymes hydrolysing a complex of polysaccharides and, finally, active and resistant microorganisms which can effect ethanol fermentation. Figure 1 shows a general diagram of the ethanol fermentation of biomass.

Pretreatment is of key importance for ensuring a high output of monosaccharides from the polysaccharides present in the material under treatment. Without the pretreatment stage, hydrolysis efficiency of less than 20% can be achieved, while pretreatment can increase it to 90% and above (Balat 2011). Effective pretreatment should meet several criteria: it should ensure effective separation of lignin from cellulose, increase the percentage of amorphous cellulose, ensure higher porosity of substrates, eliminate the loss of sugars, limit formation of inhibitors and minimise the cost of energy. Physical, physicochemical, chemical and biological pretreatment is conducted in order to increase the efficiency of energy production from biomass. It is important to establish the technological conditions which ensure that the conditioning process will be efficient, cost-effective and safe. If the treatment is not sufficiently effective, it can result in the formation of toxic compounds which inhibit the metabolism of methanogenic bacteria (Jönsson and Martín 2016; Sindhu and Pandey 2016; Behera et al. 2014).

To increase the productivity of ethanol fermentation, microwave pretreatment was tested in this study. Elucidation and understanding of the mechanism of the effect of microwave radiation on structural components of plants, mainly on the structure of polysaccharide complexes, is necessary to improve the efficiency of the conversion of biomass to energy. According to the literature reports, low-temperature (< 200 °C) microwave treatment can increase the energy value of biomass. The process efficiency in low-temperature microwave treatment depends largely on the type of material under treatment, its physical parameters, structure, conductivity and dielectric properties. In conventional thermal treatment, heat is supplied by convection and conduction; when microwave radiation is applied, energy is supplied directly to the material being heated. The application of microwave technology has a lot of potential benefits; microwaves pass through the material and they accumulate energy and generate heat in the whole volume of a material. Using microwave energy reduces the duration of heating a material, allows for process control and improves the energy efficiency. Budarin et al. (2009) studied the microwave-aided process of cellulose decomposition and found that the interaction between cellulose and microwave radiation is very intense and has a decisive effect on the decomposition of cellulose molecules. These interactions during low-temperature microwave treatment result in the formation of products which require much higher temperatures when conventional heating is applied (Budarin et al. 2009).

A comparison of microwave and conventional heating in a number of biological reactions has revealed various behaviours and properties which—as scientists argue—are proof of the nonthermal effects of microwave radiation. The difference between thermal and nonthermal effects lies in that an effect is regarded as thermal if it can be induced not only by microwave field but also by heat generated in a different way; an effect is nonthermal if it is induced by radiation of such intensity that the temperature increase it causes is negligible or if the effect cannot be achieved by increasing the temperature in a different manner (Dębicki and Styłba 2010).

Considering the effects of changes of lignocellulosic complex ultrastructure, microwave-based heating can be an alternative to conventional heating (Deng et al. 2015). Microwave radiation delignifies and partly removes hemicellulose and increases hydrolysis of sugars. Conventional heating is based on surface heat exchange; with microwaves, heat is generated by the electromagnetic field. Microwave radiation destroys cellulose by molecular collisions caused by dielectric polarisation. The advantages of this method include the short duration of the process, high selectivity and a smaller amount of the energy supplied compared with conventional heating (Hendriks and Zeeman 2009; Balat 2011). Cheng et al. (2008) improved properties of the coal water slurry (CWS) property made from Chinese Shenhua coal with high inherent moisture and oxygen contents, microwave irradiation and thermal heat. They proved that the minimum unit energy consumption of 0.115 kWh/kg and electricity cost of 4.6 USD/ton for CWS concentration promotion by 1% are obtained at 420 W for 20 s in the microwave oven. The unit energy consumptions for CWS concentration promotion and inherent moisture removal by thermal heat are, respectively, 214 and 22.5 times higher than those by microwave irradiation, while the energy use efficiencies are on the converse (Cheng et al. 2008).

The aim of this study was to determine the effect of thermal hydrolysis on degradation of polysaccharides contained in biomass of cyanobacteria *Arthrospira platensis* and to assess the effectiveness of ethanol production from preconditioned biomass. The effect of microwave-based and conventional heating on bioethanol production was compared.

## Study methodology

### Study material

Biomass of *Arthrospira platensis* obtained by the authors in tubular photobioreactors at the Department of Environmental Engineering of the University of Warmia and Mazury was used as the study material. The biomass was obtained under autotrophic conditions with a standard culturing medium (*Spirulina* medium, Aiba and Ogawa 1977). The characteristics of the biomass are provided in Table 1.

**Table 1:** Characteristics of biomass of *Arthrospira platensis*

Parameter	Unit	Value
Dry mass	(% f.m.)	5.9
Organic dry mass	(% f.m.)	83.2
Mineral dry mass	(% f.m.)	16.8
Protein	(% f.m.)	71.5
Sugars	(% f.m.)	18.9
Fats	(% f.m.)	11.1

### Study concept

The experiment was divided into two stages; in stage I, the possibility of obtaining fermentable sugars by hydrothermal treatment of the substrate was tested. Stage II involved an assessment of the effectiveness of the pretreatment methods to produce bioethanol in alcohol fermentation.

#### Stage I

The first stage of the study, with pretreatment, involved simultaneous testing of two variants of heating: microwave-based and conventional; each variant of the experiment was tested in three series at a different process temperature. The hydrothermal pretreatment was conducted for 20 min in both heating options at 100 °C, 125 °C and 150 °C. The temperature range has been selected based on literature data, from which it follows that if a temperature of 130–180 °C is used during the initial preparation, lignin and hemicelluloses begin to decompose. Literature data should advise you to avoid temperatures above 250 °C during the heat treatment process. The correctness of the selected temperature variants is confirmed not only by literature reports but also by the authors’ previous research. They showed an increase in the efficiency of hydrothermal treatment in proportion to the increase in process temperature in the range of 100 to 150 °C. Increasing the processing temperature to 180 °C did not result in a statistically significant increase in the amount of glucose released, regardless of the type of substrate heating used. In all tested temperature variants, a statistically significant increase in the hydrolysis efficiency along with the time extension of the process was observed in the range of 10 to 20 min. At longer durations of 25, 30 and 40 min, the increase in the amount of glucose released was not statistically significant, both in microwave and conventional heating. On the basis of the results obtained, the parameters of conducting the experiments were selected.

Thermohydrolysis was conducted in two heating technologies. The hydrothermal microwave treatment was carried out with the Mars-Solvent Extraction system, manufactured by CEM, with an adjustable output power of up to 1600 W and a microwave frequency of 2.45 GHz. Samples could be digested in the Mars system at temperatures of up to 300 °C. The experiment was conducted in EasyPrep Teflon vessels with a volume of 115 cm^3^. Thermohydrolysis by the conventional heating method was carried out in original thermoreactors designed by the authors. The thermoreactors were made of acid-resistant steel, and their dimensions were the same as those of EasyPrep vessels used in microwave-induced sample digestion. Conventional heating of the plant material was done with a four-stand Laboplay O420E oil bath, in which silicon oil was used as the heating agent. The conditions during the thermohydrolysis—both with conventional and microwave-based heating—were the same. The time of heating up and the temperature of thermohydrolysis were controlled to achieve the identical process parameters.

The following analytical procedures were applied to establish the best parameters of thermohydrolysis:

- Total glucose assay with a YSI biochemical analyser
- Total phenolic compounds assay with a Hach Lange—LCK 345 reagent kit

Samples for the assays were prepared by filtering the dissolved plant material through a membrane filter with a pore diameter of 1.2 μm by the method according to Hach (LCK 914).

#### Stage II

Alcohol fermentation was conducted with an industrial yeast strain used in the industrial production of ethanol: *Saccharomyces cerevisiae* As4. Inoculum of yeast *S. cerevisiae* was done with liquid YPG medium: 10 g of yeast extract, 20 g of peptone, 20 g of glucose and 20 g agar dissolved in 1000 cm^3^ of distilled water. The pH was brought to 5.2 by adding 1 M HCl and controlled by a universal pH meter.

Five cubic centimetres of sterile distilled water was transferred into a tube with a yeast culture on YPG, and washout was performed. The cell suspension obtained in the process was introduced to a conical flask with 100 cm^3^ of liquid YPG medium and incubated for 24 h in a shaker at a temperature of 30 °C.

Alcohol fermentation was conducted in an SHF (separate hydrolysis and fermentation) system. After hydrolysis, the substrate was fermented. Stage II involved testing all variants of the experiment tested at stage I.

The inoculum of the appropriate yeast strain was added to the prepared medium (substrate after pretreatment). Fermentation was conducted for 72 h under anaerobic conditions, at an appropriate temperature for the yeast strain, i.e. at 38 °C.

The content of ethanol in the medium was determined after the fermentation was completed. This was done by the method using Hach Lange’s LCK 300 reagents.

Statistical analysis of the results was done with the STATISTICA 12.0 PL; a one-way analysis of variance (ANOVA) was conducted in order to check the significance of the differences between variables. A RIR Tukey test was conducted in order to determine the significance of differences between the variables under analysis. The level of significance of *p* = 0.05 was adopted in the tests.

## Results

The amount of produced glucose and phenolic compounds were analysed to assess the effectiveness of the pretreatment process.

**Fig. 2:**
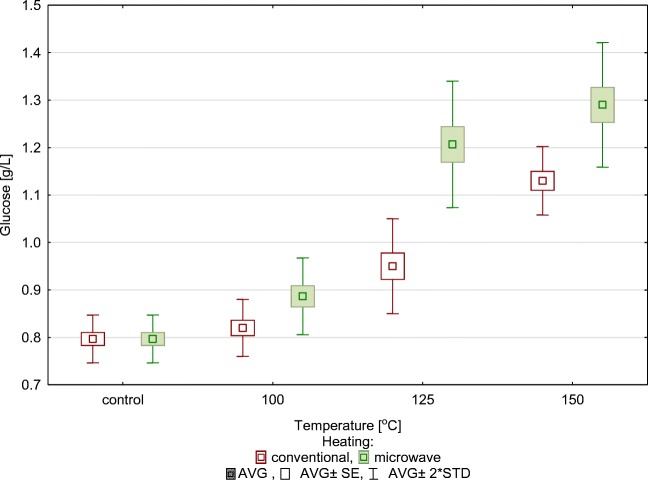
Concentration of glucose in relation to temperature and type of thermohydrolysis

The amount of glucose released to the solution (Fig. 2) was used to conclude that 150 °C is the most effective pretreatment temperature of the tested values of 100, 125 and 150 °C. This conclusion applies both to microwave and conventional heating. These results show that microwave heating is more effective than traditional heating. Comparing samples after thermohydrolysis to a control sample shows that applying the microwave technology at 100 °C increased the amount of produced glucose by 10%, at 125 °C by 48% and at 150 °C by 62%. In an analogous series, the application of conventional heating brought about an increase in the amount of produced glucose by 2.5%, 19% and 40%.

**Fig. 3:**
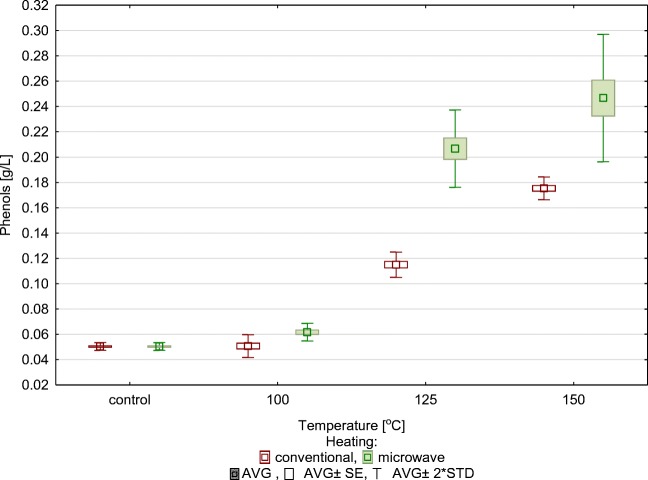
Concentration of phenolic compounds in relation to temperature and type of thermohydrolysis

There is a risk of formation of inhibitors of alcohol fermentation during the process of thermohydrolysis; taking this into consideration, the amount of phenolic compounds formed following the thermal treatment was determined (Fig. 3.). The control sample contained 0.05 g/l of phenols, heating it up to 150 °C brought about the greatest increase in the analysed compounds when conventional heating as applied: the amount of phenols increased to 0.17 g/l; it increased to 0.25 g/l as a result of microwave heating.

**Fig. 4:**
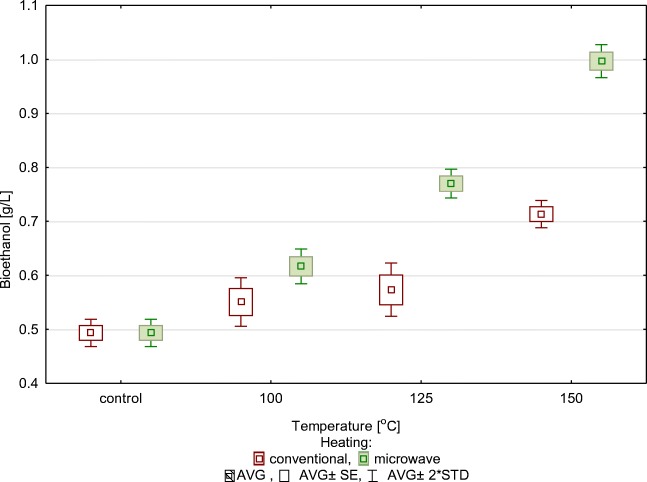
Concentration of bioethanol in relation to the type of thermohydrolysis

These results show that an increase in the concentration of phenolic compounds did not inhibit alcohol fermentation, but it was correlated with the concentration of produced glucose (Fig. 4). The content of ethanol in the medium—“mash”—was determined after fermentation was completed (72 h). Fermented samples were prepared at 100 °C, 125 °C and 150 °C. The control substrate yielded 0.49 g/l of bioethanol, the alcohol concentration in the conventionally preheated sample increased by 45%, i.e. to 0.71 g/l, and the microwave-heated sample contained the highest concentration of alcohol (0.97 g/l), which is more by 98% than in the control mash and more by 37% than in the conventionally heated sample.

## Discussion

The literature provides examples of application of microwaves as a conditioning factor in alcohol fermentation. Substrate exposure to microwave radiation in various temperature ranges has shown that microwaves increase solubilisation of substrates and break the network of exopolysaccharides, thereby speeding up chemical processes (Ahn et al. 2009).

Klein et al. (2016) applied microwave radiation to increase the production of bioethanol from leaves of sacred fig. The authors applied simultaneous microwave chemical treatment with hydrochloric acid as the reagent used in the experiment. They compared the results with conventionally heated samples. They tested saccharification of 1 g of the substrate with 20 ml of HCl as a function of acid concentration, time and temperature of exposure to microwave radiation. They observed the most satisfying results after adding 1 M HCl to the sacred fig leaves, followed by microwave heating for 8 min at temperatures increasing from 80 to 110 °C. The application of microwave radiation increased glucose production by up to 246% compared with conventionally heated samples. After the pretreatment, alcohol fermentation of the hydrolysate was conducted with *Saccharomyces cerevisiae*; the maximum ethanol recovery was 3% (*w*/*w*) of the dry weight of the substrate used (Klein et al. 2016). Microwave thermohydrolysis conducted by those authors increased the amount of produced glucose by 62%, which translated into an increase in bioethanol production by 98% compared with the control sample.

Zhu et al. (2006) demonstrated that simultaneous treatment of wheat straw with alkalis and with electromagnetic radiation results in smaller loss of sugars and a higher degree of the substrate hydrolysis (Zhu et al. 2006). Microwave heating as a supplementary method to pretreatment with ammonia (28% of the dry weight of substrate) sorghum residue in bioethanol production was applied by Chen et al. (2012). The best results relative to the amount of produced glucose of 4.2 g per 10 g_d.m._ and ethanol yield of 2.1 g per 10 g_d.m._ were obtained following treatment at 130 °C for 60 min. The best degree of delignification—46%—was recorded at 160 °C. The concentrations of furfural, organic acids and glycerol were low, owing to which they did not inhibit the activity of enzymes in hydrolysis or yeast in saccharide fermentation (Chen et al. 2012). The amount of phenolic compounds produced during the process of thermohydrolysis did not have an inhibitory effect. Despite a considerable increase in the amount of phenols (from 0.05 in the control sample to 0.25 g/l in the sample following microwave thermohydrolysis), the efficiency of fermentation was much higher after pretreatment than in the control sample with no pretreatment.

Hu and Wen (2008) applied microwave thermal pretreatment before enzymatic hydrolysis of switchgrass. They showed that when the substrate is incubated for 2 h in solution of NaOH before the thermal treatment by means of microwave radiation, 99% of the theoretical efficiency of the substrate saccharification can be achieved. Incubation of sorghum at 20 °C for 120 min followed by treatment of the substrate with microwave radiation for 30 min at 190 °C yielded 58.5 g glucose and xylose from 100 g of biomass (Hu and Wen 2008).

Similar experiments were conducted by Shengdong et al. (2006), who tested the effect of a combination of microwave and alkaline treatment on the hydrolysis of wheat straw. They compared the findings of their study with the results of treatment with NaOH without heating the substrate. They compared the share of both fractions of lignocellulose following both types of treatment; the content of cellulose was 79.6/73.5%, lignin 5.7/7.2%, and hemicellulose 7.8/11.2% for the microwave alkaline and alkaline treatment, respectively. The experiment demonstrated not only a larger amount of lignin and cellulose removed when the combined method was applied but also a higher degree of the substrate hydrolysis than that observed when only the alkaline treatment was applied without microwave radiation (Shengdong et al. 2006).

Meinita et al. (2013) examined production of bioethanol from red algae: *Gelidium amansii*, *Gracilaria tenuistipitata*, and *Gracilariopsis chorda*. The algae were hydrolysed at 130 °C for 15 min with 0.2 M H_2_SO_4_ and subsequently fermented at 30 °C; the distillate contained from 0.5 to 0.83 g/l of ethanol (Meinita et al. 2013). In own studies, the microwave-heated sample contained the highest concentration of alcohol (0.97 g/l). Wu et al. conducted fermentation of algae of *Gracilaria sp.* and produced ethanol with the yeast *Saccharomyces cerevisiae* Wu-Y2; they obtained maximally 4.72 g/l of ethanol from 11.85 g/l of glucose (Wu et al. 2014).

Zhao et al. proved that treating Chlorella sp. with ultrasounds increased the extraction of sugars. The ultrasonic treatment was carried out using various powers (600, 800 and 1000 W) and various pretreatment times (30, 60 and 90 min). Maximum glucose yield 36.8 g/100 g_d.w_. was gained at the highest power of 1000 W at 80 min (Zhao et al. 2013).

Kim et al. proved that the physical pretreatment of seaweed *Gelidium amansii* in autoclave at 120 °C at different time intervals—20, 40, 60, 80, and 60 min—caused an increase in glucose by 59% as a result of the control (Kim et al. 2015). This result is similar to the result obtained by the authors of the study, in which a 60% increase in glucose secretion was noted.

In studies, the highest level of bioethanol was obtained during fermentation of the substrate after 20 min, pretreatment at 150 °C. The microwave-heated sample contained the highest concentration of alcohol (0.97 g/l), which is more by 98% than in the control mash and more by 37% than in the conventionally heated sample. The literature presents different values of bioethanol depending on the type of applied microalgae or macroalgae and pretreatment methods. Bioethanol yields from *Chlorella vulgaris* after acid pretreatment 11.66 g/l and after enzymatic pretreatment 4.27 g/l. Bioethanol yields from *Chlorococcum infusionum* after alkaline pretreatment 0.26 g/g and *Scenedesmus obliquus* after acid and autoclave pretreatment 0.202 g/g. Bioethanol yields from macroalgae *Gracilaria cliftonii* after acid and enzyme pretreatment 4.27 g/l (Sirajunnisa and Surendhiran 2016).

## Conclusions

The application of microwave heating with a view to hydrothermal disintegration of cyanobacteria *Arthrospira platensis* helped to achieve higher efficiency of glucose release and production of ethanol compared with conventional heating. The application of microwave technology increased the amount of produced glucose by a maximum of 62% compared with a sample with no pretreatment. The results have shown that 150 °C was the most beneficial for the thermohydrolysis process, both in the microwave and conventional heating. The results show that the increased concentration of phenolic compounds did not inhibit alcohol fermentation; the control mash yielded 0.49 g/l of bioethanol, the alcohol concentration in the conventionally preheated sample increased by 45% (to 0.71 g/l), and the mash in the microwave-heated sample contained the highest concentration of alcohol (0.97 g/l), which is 98% more than in the control mash and 37% more than in the conventionally heated sample.
